# Leydig and Sertoli cell function in individuals with genital ambiguity, 46,XY karyotype, palpable gonads and normal testosterone secretion: a case-control study

**DOI:** 10.1590/1516-3180.2021.0042.R1.08062021

**Published:** 2022-02-07

**Authors:** Guilherme Guaragna-Filho, Antônio Ramos Calixto, Anna Beatriz Lima do Valle Astur, Georgette Beatriz de Paula, Laurione Cândido de Oliveira, André Moreno Morcillo, Ezequiel Moreira Gonçalves, Maricilda Palandi de Mello, Andrea Trevas Maciel-Guerra, Gil Guerra-Junior

**Affiliations:** I MD, PhD. Adjunct Professor, Department of Pediatrics, School of Medicine, Universidade Federal do Rio Grande do Sul (UFRGS), Porto Alegre (RS), Brazil.; II PhD. Researcher, Laboratory of Investigation in Metabolism and Diabetes (LIMED), Universidade Estadual de Campinas (UNICAMP), Campinas (SP), Brazil.; III MD. Attending Physician, Interdisciplinary Group for Study of Sex Determination and Differentiation (GIEDDS), School of Medical Sciences, Universidade Estadual de Campinas (UNICAMP), Campinas (SP), Brazil.; IV MD, MSc. Attending Physician, Interdisciplinary Group for Study of Sex Determination and Differentiation (GIEDDS), School of Medical Sciences, Universidade Estadual de Campinas (UNICAMP), Campinas (SP), Brazil.; V PhD. Researcher, Laboratory of Physiology, Clinical Hospital, Universidade Estadual de Campinas (UNICAMP), Campinas (SP), Brazil.; VI MD, PhD. Associate Professor, Department of Pediatrics, School of Medical Sciences, Universidade Estadual de Campinas (UNICAMP), Campinas (SP), Brazil.; VIII PhD. Adjunct Professor, Growth and Development Laboratory, Center for Investigation in Pediatrics (CIPED), School of Medical Sciences, Universidade Estadual de Campinas (UNICAMP), Campinas (SP), Brazil.; VIII PhD. Researcher, Center of Molecular Biology and Genetic Engineering (CBMEG), Universidade Estadual de Campinas (UNICAMP), Campinas (SP), Brazil.; IX MD, PhD. Full Professor, Interdisciplinary Group for Study of Sex Determination and Differentiation (GIEDDS), School of Medical Sciences, Universidade Estadual de Campinas (UNICAMP), Campinas (SP), Brazil.; X MD, PhD. Full Professor, Interdisciplinary Group for Study of Sex Determination and Differentiation (GIEDDS), School of Medical Sciences, Universidade Estadual de Campinas (UNICAMP), Campinas (SP), Brazil.

**Keywords:** Inhibin B [supplementary concept], Disorders of sex development, Sertoli cells, Leydig cells, DSD, Ambiguous genitalia, INSL3, AMH

## Abstract

**BACKGROUND:**

Because normal male sexual differentiation is more complex than normal female sexual differentiation, there are more cases of disorders of sex development (DSDs) with 46,XY karyotype that have unclear etiology. However, Leydig and Sertoli cell markers are rarely used in distinguishing such individuals.

**OBJECTIVES:**

To evaluate the function of Leydig and Sertoli cells in individuals with genital ambiguity, 46,XY karyotype, palpable gonads and normal testosterone secretion.

**STUDY DESIGN AND SETTING:**

Case-control study with 77 patients, including eight with partial androgen insensitivity syndrome, eight with 5α-reductase deficiency type 2 (5ARD2) and 19 with idiopathic 46,XY DSD, and 42 healthy controls, from the Interdisciplinary Study Group for Sex Determination and Differentiation (GIEDDS), at the State University of Campinas (UNICAMP), Campinas, Brazil.

**METHODS:**

Baseline levels of gonadotropins, anti-Müllerian hormone (AMH), inhibin B, insulin-like 3 (INSL3), testosterone and dihydrotestosterone in cases, and AMH, inhibin B, and INSL3 levels in controls, were assessed.

**RESULTS:**

There was no significant difference in age between cases and controls (P = 0.595). AMH and inhibin B levels were significantly lower in cases than in controls (P = 0.031 and P < 0.001, respectively). INSL3 levels were significantly higher in cases than in controls (P = 0.003). Inhibin B levels were lower in 5ARD2 patients (P = 0.045) and idiopathic patients (P = 0.001), in separate comparisons with the controls.

**CONCLUSION:**

According to our findings, we can speculate that inhibin B levels may be used to differentiate among DSD cases.

## INTRODUCTION

Ambiguous genitalia are the most complex clinical manifestation of disorders of sex development (DSDs), an umbrella term that is used for congenital conditions characterized by atypical chromosomal, gonadal or anatomical development.^1^ Because normal male sexual differentiation involves more genetically determined and hormonal events than those in normal female sexual differentiation, DSDs with 46,XY karyotype present greater etiological complexity.

Among the main etiologies of patients with genital ambiguity, 46,XY karyotype and normal testosterone secretion, partial androgen insensitivity syndrome (PAIS) (OMIM #312300) potentially presents with clinical features indistinguishable from those of other etiologies, particularly those of 5α-reductase type 2 deficiency (5ARD2) (OMIM #264600).^2^ Both diagnoses are solely confirmed on the basis of molecular alterations in specific genes, which is a costly procedure and thus conducted at only a few centers.^3^ Routine measurement of the function of Leydig cells, including testosterone and dihydrotestosterone (DHT) levels, is not always effective for differentiating PAIS from 5ARD2.^3,4^

There is a lack of data regarding insulin-like 3 (INSL3), which is also produced by Leydig cells, in relation to management of DSDs.^4^ In some studies, high levels of anti-Müllerian hormone (AMH), a marker for Sertoli cells, were observed in patients with androgen insensitivity.^5–7^ However, in patients with 5ARD2, low levels have been reported in only two studies.^8,9^ Inhibin B is a good marker for Sertoli cells and gonadal function; nevertheless, data on cases of androgen insensitivity and 5ARD2 remain limited.^4^ Moreover, the role of these Leydig and Sertoli cell markers in patients with idiopathic 46,XY DSDs and genital ambiguity remains unclear.

## OBJECTIVE

The aim of this study was to evaluate the function of Leydig cells (testosterone, dihydrotestosterone [DHT] and INSL3) and Sertoli cells (AMH and inhibin B) in patients with 46,XY DSDs and genital ambiguity.

## METHODS

### Patients and control group

The inclusion criteria were palpable gonads (in the scrotal and/or inguinal region, bilaterally), 46,XY karyotype and normal testosterone secretion during etiological investigation of genital ambiguity. The *AR* (OMIM *313700), *SRD5A2* (OMIM *607306) and *NR5A1* (OMIM *184757) genes were sequenced in all patients included in this study. All patients with PAIS had *AR* mutations, all patients with 5ARD2 had *SRD5A2* mutations and patients with idiopathic 46,XY DSDs had no mutations in these three genes sequenced.

The patients were selected from a sample of 408 patients first reported in 2016,^10^ who had been diagnosed with DSDs between 1989 and 2016 by the Interdisciplinary Study Group for Sex Determination and Differentiation (GIEDDS) at the Universidade Estadual de Campinas (UNICAMP), Campinas (SP), Brazil. In this sample, among 189 individuals with 46,XY karyotype and both testicles, 107 patients had normal testosterone secretion. Among these 107 patients, 10 were diagnosed with PAIS (five prepubertal, four pubertal and one gonadectomized), 20 with 5ARD2 (five prepubertal, eight pubertal and seven gonadectomized) and 77 with idiopathic 46,XY DSDs (15 prepubertal, 61 pubertal and one gonadectomized). Furthermore, among these 107 patients, 98 met the inclusion criterion (not gonadectomized) for this study. However, only 54 continued with routine follow-up at the DSD outpatient clinic and, among these, 35 (65%) provided consent to participate in this study.

The control group comprised males aged three months to 40 years, including in-hospital patients, postgraduate students and their family members, with no comorbidities resulting in altered testicular function. The exclusion criteria for the control group were the following: birth weight less than 2500 g, previous history of genital ambiguity, hypospadias, varicocele, unilateral or bilateral cryptorchidism, infections or any disorder in the testicles, moderate-to-severe traumatic lesions in the testicles, testicular neoplasia, adrenal disease and use of testicular function-altering or gonadal axis-altering drugs.

This study was approved by the Research Ethics Committee of our institution (protocol number CEP: 434/2006, approved on August 26, 2014) and was conducted in accordance with the principles of the Declaration of Helsinki.

### Clinical evaluation

Upon recruitment, patients were clinically evaluated with regard to the following variables: age (in months), weight (in kg), height (in cm) and body mass index (BMI; in kg/m^2^). These values were converted to z-scores using the NCHS 2000 data. Moreover, the patients’ stage of puberty was assessed, and the patients were then classified as pubertal (Tanner stage ≥ 2) or pre-pubertal (Tanner stage 1). Furthermore, the following data were obtained from medical records: birth weight (in g), birth length (in cm) and features of the genitalia at initial presentation. The grade of masculinization of the genitalia was determined on the basis of the external masculinization score (EMS), in accordance with the method described by Ahmed et al.^11^

### Laboratory evaluation

Karyotyping was performed at the cytogenetics laboratory at our institution, with a minimum count of 32 sets of metaphases. Only patients with a homogeneous 46,XY karyotype were included in the study.

For hormonal evaluation, the baseline levels of luteinizing hormone (LH), follicle-stimulating hormone (FSH), testosterone, DHT, AMH, inhibin B and INSL3 were determined for all patients. Furthermore, all prepubertal patients underwent a stimulation test using human chorionic gonadotropin (hCG) (1,500 IU/d; via intramuscular injection on three consecutive days), and testosterone and DHT levels were measured 24 hours after the last dose. Testosterone secretion was considered normal in individuals presenting a total increase in testosterone of 1.5 ng/ml after stimulation relative to baseline levels.^3,12^ In these cases, only testosterone and DHT levels after hCG administration were evaluated. Only baseline AMH, inhibin B and INSL3 levels were measured in the control group. Blood samples were collected through peripheral vein puncture, and serum was extracted via centrifugation at 2000 ×*g* for 10 minutes and was stored at -20 °C until evaluation.

### Hormonal assays

The following hormonal assays were performed: LH, electrochemiluminescence (Roche Elecsys 2010, Roche Diagnostics, Switzerland); FSH, electrochemiluminescence (Roche Elecsys 2010); testosterone, electrochemiluminescence (Roche Elecsys 2010); DHT, enzyme immunoassay (ELISA, DIAsource, Belgium); AMH, enzyme immunoassay (AMH Gen II ELISA, Beckman-Coulter, Pasadena, CA, United States); inhibin B, enzyme immunoassay (Inhibin B ELISA RUO, Ansh Labs, Webster, TX, United States); and INSL3, enzyme immunoassay (Insulin-Like Protein 3 ELISA, Cloud-Clone Corp., China).

### Statistical analysis

The data were analyzed using the Statistical Package for the Social Sciences (SPSS) software version 21.0 (SPSS Inc., Chicago, IL, United States). The Shapiro-Wilk test was performed to verify data normality. Because most data were not normally distributed, we performed nonparametric tests. To compare variables between two groups (cases versus controls and pre-pubertal versus pubertal groups), the Mann-Whitney U test for independent samples was performed. To compare groups in accordance with the diagnoses (PAIS, 5ARD2 and idiopathic), the Kruskal-Wallis test was performed, followed by a multiple-comparisons test with Bonferroni adjustments to determine the differences among the groups, if necessary. Additionally, multivariate linear regression analysis using the stepwise method was performed to verify the influence of the dependent variables (age, puberty, weight, height, BMI, weight z-score, height z-score, BMI z-score, EMS, birth weight and birth length), with regard to explaining the variation in the expression of INSL3, inhibin B and AMH markers. The puberty variable was analyzed as a dummy (0 = no puberty and 1 = puberty). The linear regression parameters were presented as values of the unstandardized coefficients: beta (β) ± standard error and as values of the adjusted explanation coefficient (r^2^). The significance level was set at 5% (P < 0.05).

## RESULTS

Among the 35 patients included in the study, eight had PAIS (five prepubertal and three pubertal), eight had 5ARD2 (three prepubertal and five pubertal) and 19 were idiopathic (13 prepubertal and six pubertal). The baseline clinical and laboratory data of all the patients included are summarized in **Table 1**. The control group comprised 42 individuals aged 137.7 ± 125.9 months (mean ± standard deviation; median = 123 months, minimum = 3 months, and maximum = 408 months). Age (in months) at the time of the current assessment was not significantly different between cases and controls (Mann-Whitney U test; P = 0.595).

**Table 1.** Baseline clinical and laboratory data of the 35 cases with 46,XY DSDs

	PAIS (n = 8)			5ARD2 (n = 8)			Idiopathic (n = 19)			P-value
	Median	Min	Max	Median	Min	Max	Median	Min	Max	
**Age (months)**	92	46	430	196	5	469	98	9	217	0.509
**Weight (kg)**	29	16	91	54	9	90	27	7	65	0.260
**Height (cm)**	130	102	169	168	68	180	129	68	169	0.257
**BMI (kg/m^2^)**	16.6	15.3	32.0	19.6	18.0	27.6	16.3	12.3	24.5	0.084
**Weight (z-score)**	**1.5**	**−0.02**	**2.4**	1.0	-1.5	1.5	**−0.2**	**−3.1**	**1.4**	0.003*
**Height (z-score)**	**0.4**	**−0.9**	**2.6**	0.6	-1.3	1.0	**−0.9**	**−3.1**	**0.9**	0.024*
**BMI (z-score)**	0.4	-1.2	3.0	1.0	-0.9	1.5	0.3	-3.6	1.3	0.375
**EMS**	6	5	9	4	2	9	6	5	9	0.057
**Birth weight (g)**	2910	1510	3950	2900	1500	3500	2290	700	3600	0.142
**Birth length (cm)**	46	42	52	**49**	**43**	**53**	**44**	**31**	**49**	**0.033**^**†**^
**FSH (IU/l)**^**a**^	1.48	0.67	6.44	7.60	0.25	35.07	0.96	0.42	16.28	0.320
**LH (IU/l)**^**a**^	0.15	0.10	14.71	6.22	0.10	16.93	0.14	0.10	6.17	0.169
**Testosterone (ng/ml)**^**b**^	2.09	1.51	8.72	5.08	1.83	10.97	3.18	1.53	7.79	0.317
**DHT (pg/ml)**	103.10	52.70	674.40	362.65	37.60	1652.20	148.10	25.90	1454.80	0.472
**T/DHT**	21.0	7.5	30.0	14.7	6.6	54.0	19.8	2.5	59.1	0.697

DSD = disorders of sex development; PAIS = partial androgen insensitivity syndrome; 5ARD2 = 5α-reductase type 2 deficiency; Min = minimum; Max = maximum; BMI = body mass index; EMS = external masculinization score; FSH = follicle-stimulating hormone; LH = luteinizing hormone; DHT = dihydrotestosterone; T = testosterone. ^a^Baseline levels of all subjects; ^b^Post-human chorionic gonadotropin (hCG) levels in prepubertal subjects, baseline levels in pubertal subjects; *Significant differences between PAIS and 5ARD2 groups (post-hoc adjustment through Bonferroni test); ^†^Significant differences between 5ARD2 and idiopathic groups (post-hoc adjustment through Bonferroni test).

Regarding baseline levels among patients, no significant difference (Kruskal-Wallis test) was observed among the three subgroups with regard to age (months) at the current evaluation (P = 0.509), weight (P = 0.260), height (P = 0.257), BMI (P = 0.084), z-score of BMI (P = 0.375), EMS at initial presentation (P = 0.057), birth weight (P = 0.142), FSH (P = 0.320), LH (P = 0.169), testosterone (P = 0.122), DHT (P = 0.485), and testosterone-DHT ratio (T/DHT) (P = 0.989). However, significant differences (Kruskal-Wallis test) in the z-scores of current weight (P = 0.003) and height (P = 0.024) were observed, such that both of these were lower in the idiopathic group only, compared with the PAIS group. In contrast, length at birth was significantly shorter in the idiopathic group than in the 5ARD2 group (P = 0.030) (**Table 1**).

AMH levels were inversely proportional to age, with a moderate correlation between cases (r = −0.68; P < 0.0001) and controls (r = −0.83; P < 0.0001). Subgroup analysis revealed that this age-based correlation was strong in the 5ARD2 group (r = −0.95; P < 0.0001) and moderate in the idiopathic group (r = −0.71; P = 0.001), but not significant in the PAIS group (r = −0.33; P = 0.420). Furthermore, AMH was positively correlated with inhibin B only in the idiopathic group (r = 0.56; P = 0.039): there was only a positive correlation between these two hormones in this group, among all the groups (r = 0.55; P = 0.002). On the other hand, this correlation was not observed in relation to the 5ARD2 (r = 0.061; P = 0.148) and PAIS (r = 0.19; P = 0.651) subgroups. Serum AMH levels were significantly lower in cases than in the control group (P = 0.031). Nonetheless, subgroup comparisons with the control group and among the groups did not reveal any significant differences in AMH levels (**Table 2**).

**Table 2.** Comparison of anti-Müllerian hormone, inhibin B and INSL3 levels between cases and controls

	Cases					Controls					P-value
	n	Median	IQR	Min	Max	n	Median	IQR	Min	Max	
**Age (months)**	35	100.00	135.00	9.00	469.00	42	123.00	162.25	3.00	408.00	0.595
**AMH (pMol/l)**	35	243.51*	228.52	8.96	295.85	42	292.90	314.07	20.06	420.55	< 0.001
**Inhibin B (pg/ml)**	29	65.14*	106.86	14.18	381.37	42	151.59	149.86	57.11	926.84	0.003
**INSL3 (ng/ml)**	32	0.35*	0.03	0.33	3.51	31	0.21	0.26	0.10	1.04	0.031

INSL3 = insulin-like 3; IQR = interquartile range; AMH = anti-Müllerian hormone; Min = minimum; Max = maximum. *Significant differences, compared with the controls (independent-samples Mann-Whitney U test).

Inhibin B levels could not be evaluated in one case with 5ARD2 (aged 27 years 10 months) and in five cases in the idiopathic subgroup (aged 1 year 5 months, 6 years 6 months, 7 years 3 months, 8 years 2 months and 8 years 4 months). Inhibin B levels were evaluated in all patients in the PAIS and control groups, and no correlation with age was observed, either among cases or controls. As described above, inhibin B was positively correlated with AMH only in the idiopathic group. Inhibin B levels were significantly lower in cases than in controls (P < 0.001) (**Table 2**). Comparison of all subgroups of cases (PAIS, 5ARD2 and idiopathic) with the control group and with each other only showed significantly lower values in the 5ARD2 (P = 0.045) and idiopathic groups (P = 0.001) than in controls, using Bonferroni’s adjusted multiple comparison test (**Table 3**). No significant differences were observed in subsequent comparisons.

**Table 3.** Comparison of anti-Müllerian hormone, inhibin B and INSL3 levels between subgroups of cases and controls

	n	PAIS		n	5ARD2		n	Idiopathic		n	Control	
		Median	IQR		Median	IQ		Median	IQR		Median	IQ
**Age (months)**	8	92.0	267.7	8	196.5	259.5	19	98.0	113.0	42	123.0	162.2
**AMH (pMol/l)**	8	261.50	112.37	8	42.05	243.15	19	247.04	210.84	42	292.90	314.04
**Inhibin B (pg/ml)**	8	93.49	156.59	7	50.70*	**101.41**	14	**59.52**^†^	**81.89**	42	**151.59**	**149.86**
**INSL3 (ng/ml)**	8	0.36	0.04	7	0.35	0.05	17	0.35	0.02	31	0.21	0.26

INSL3 = insulin-like 3, PAIS = partial androgen insensitivity syndrome; 5ARD2 = 5α-reductase type 2 deficiency; IQR = interquartile range; AMH = anti-Müllerian hormone. *Significant difference (lower values), compared with the control group (post-hoc adjustment through Bonferroni test; P = 0.045); ^†^Significant difference (lower values), compared with the control group (post-hoc adjustment through Bonferroni test; P = 0.001).

INSL3 levels could not be evaluated in one case with 5ARD2 (aged 18 years), two cases in the idiopathic subgroup (aged 8 years 4 months and 18 years 1 month) and 11 controls (aged 10 months, 11 months, 1 year 7 months, 2 years 1 month, 2 years 10 months, 8 years 3 months, 14 years 1 month, 14 years 10 months, 28 years, 29 years and 34 years). INSL3 was not significantly correlated with age in any of the groups analyzed (controls: r = 0.251; p = 0.177; PAIS: r = 0.000; p = 1.00; 5ARD2: r = −0.236; P = 0.610; and idiopathic: r = −0.202; P = 0.437). Moreover, INSL3 levels were not correlated with inhibin B (controls: r = −0.327; P = 0.072; PAIS: r = −0.195; P = 0.643; 5ARD2: r = −0.213; P = 0.686; and idiopathic: r = 0.000; P = 1.00) or AMH levels (controls: r = −0.182; P = 0.328; PAIS: r = 0.053; P = 0.773; 5ARD2: r = 0.059; P = 0.900; and idiopathic: r = 0.100; P = 0.703). Intergroup comparisons revealed that INSL3 levels were significantly higher in cases than in controls (P = 0.003) (**Table 2**). Subgroup comparisons among each other and with the control group revealed higher values in the 5ARD2 and PAIS subgroups than in the control group; however, these differences were not significant (Bonferroni test) (**Table 3**).

**Table 4** shows the hormone levels in pubertal and pre-pubertal individuals in each subgroup. In pre-pubertal individuals, none of the hormone levels differed significantly among the three subgroups of etiological diagnosis (Kruskal-Wallis test, P > 0.05). However, among pubertal individuals, serum inhibin B levels (Kruskal-Wallis test, P = 0.040) and AMH levels (Kruskal-Wallis test, P = 0.036) were significantly higher in the PAIS group than in the 5ARD2 and idiopathic groups.

**Table 4.** Comparison of laboratory data among the three subgroups of cases, according to the presence or absence of puberty

	Partial androgen insensitivity syndrome			5α-reductase type 2 deficiency			Idiopathic		
	Puberty								
	Yes (n = 3)	No (n = 5)	P	Yes (n = 5)	No (n = 3)	P	Yes (n = 6)	No (n = 13)	P
**LH (IU/l)**	7.65 ± 3.76	0.10 ± 0.02	0.036*	11.03 ± 2.07	0.10 ± 0.30	0.036*	4.52 ± 0.61	0.10 ± 0.39	0.001*
**FSH (IU/l)**	1.91 ± 1.57	1.16 ± 0.22	0.143	14.05 ± 5.18	0.91 ± 0.39	0.036*	9.19 ± 2.48	0.88 ± 0.60	0.001*
**Testosterone (ng/ml)**	7.95 ± 2.10	1.82 ± 0.26	0.143	5.32 ± 1.20	2.03 ± 2.71	0.393	3.57 ± 0.80	2.06 ± 0.47	0.127
**DHT (pg/ml)**	571.90 ± 185.36	86.80 ± 33.89	0.393	496.60 ± 246.36	60.30 ± 65.68	0.036*	566.75 ± 167.49	113.80 ± 18.81	0.001*
**T/DHT**	15.25 ± 4.89	24.71 ± 4.14	0.571	8.78 ± 2.06	41.02 ± 6.83	0.036*	6.95 ± 1.96	23.45 ± 4.40	0.001*
**AMH (pMol/l)**	169.65 ± 61.28	279.49 ± 21.68	0.786	31.92 ± 3.40	274.82 ± 6.41	0.036*	42.30 ± 10.83	253.43 ± 18.86	0.001*
**Inhibin B (pg/ml)**	219.51 ± 18.80	81.30 ± 11.73	0.036^*^	42.32 ± 25.93	57.11 ± 109.58	0.400	50.34 ± 25.87	61.53 ± 24.45	0.662
**INSL3 (ng/ml)**	0.36 ± 0.02	0.35 ± 0.01	1.000	0.36 ± 0.01	0.35 ± 0.02	1.000	0.34 ± 0.01	0.35 ± 0.26	0.234

LH = luteinizing hormone; FSH = follicle-stimulating hormone; DHT = dihydrotestosterone; T = testosterone; AMH = anti-Müllerian hormone; INSL3 = insulin-like 3.

In the stepwise multivariate linear regression analysis, we observed that among all the variables analyzed, the weight z-score (β = 0.006 ± 0.003; P = 0.049) significantly explained 12% of the INSL3 variations (r^2^ = 0.121); the BMI z-score (β = −27.342 ± 12.972; P = 0.047) significantly explained 13% of the variation in inhibin B (r^2^ = 0.130); and body weight (β = −3.812 ± 0.499; P < 0.001) significantly explained 68% of the variation in AMH (r^2^ = 0.680).

## DISCUSSION

To our knowledge, our study was the first to evaluate the function of Leydig and Sertoli cells in patients with genital ambiguity, 46,XY karyotype, palpable gonads and normal testosterone secretion. This particular group of DSDs was selected because of the difficulty in distinguishing such patients, especially those with 5ARD2 and PAIS, which comprise most 46,XY DSDs, based on clinical and laboratory findings before puberty.^2,10^ Thus, the present study provides promising results, especially with regard to inhibin B levels.

Our results show that the severity of external genital ambiguity, evaluated on the basis of the EMS, did not differ among these three subgroups of 46,XY DSDs with normal testosterone secretion. From a clinical viewpoint, the only difference observed was the shorter length at birth and the lower z-scores for height in the idiopathic group. These findings have already been reported previously because approximately 10%-25% of cases of 46,XY DSDs with no defined etiology are associated with intrauterine growth restriction.^13,14,15^

Furthermore, the present study confirmed that traditional methods of evaluation, such as measurement of the levels of gonadotrophins (LH and FSH) and androgens (testosterone, DHT and T/DHT), are not sufficient to differentiate among the subgroups analyzed. Currently, the recommendations indicate use of more sensitive methods, including mass spectrometry or other hormone markers, for this evaluation.^16,17^ However, Chan et al. reported that even with highly sensitive methods, androgen evaluation may be ineffective in differentiating among 46,XY DSD individuals with normal testosterone secretion.^18^

Serum AMH levels were low in all groups of 46,XY DSD individuals, relative to the control group. However, serum AMH levels were not a useful parameter for differentiating among the three subgroups of our study, in line with the findings from previous studies^5,6,8^ and with the current DSD consensus.^19^

Inhibin B, which was not indicated as a diagnostic marker in the 2016 DSD consensus,^19^ was also evaluated in this study. It has been demonstrated to be an useful parameter in evaluating DSDs, especially among individuals with 46,XY karyotype.^20,21^ The clinical significance of inhibin B is particularly evident in cases of cryptorchidism, which is a frequent clinical manifestation in patients with 46,XY DSDs with normal testosterone secretion; and in assessing fertility, which is an important aspect in DSD management.^22–26^ Studies on cryptorchidism have revealed an association between inhibin B levels and testicular volume, and have suggested that inhibin B acts as a marker for testicular recovery after treatment for cryptorchidism.^22,23^

Some studies evaluating inhibin B levels in patients with infertility have reported that a direct association between this hormone and sperm parameters was observed, which would suggest that inhibin B is also a good marker for spermatogenesis.^24,25,26^ Moreover, serum inhibin B levels are low in individuals with 46,XY gonadal dysgenesis,^27^ and in some cases, this disorder may present even with normal serum testosterone levels.^28^ In such cases, inhibin B could be used for differential diagnosis and in the prognosis for gonadal viability.

From a physiological point of view, inhibin B is useful for assessing 46,XY DSDs because it is present at measurable levels for most of an individual’s lifespan.^21,29^ In addition, this hormone is already present at measurable levels at birth, even in cord blood samples, and its level increases more rapidly during the first week of life, unlike the levels of the hormones traditionally used to assess genital ambiguities (e.g. testosterone and AMH).^29,30,31^

Furthermore, the present study showed that, similar to AMH, inhibin B levels were lower in cases than in controls. No age-related changes were observed in inhibin B levels; however, again similar to AMH, inhibin B levels were higher in pubertal patients in the PAIS group than in those in the 5ARD2 and idiopathic groups.^7^ In general, the 5ARD2 and idiopathic groups displayed lower inhibin B levels than those of the controls. This finding indicates that individuals with 5ARD2 potentially have defects in Sertoli cell maturation.^32,33^ Moreover, DHT potentially influences spermatocyte growth and differentiation,^32^ thus explaining the reduction in inhibin B levels in this group. These findings suggest that inhibin B has good potential as a biochemical marker that can differentiate patients with 5ARD2 from those with PAIS. Similar results were obtained previously among idiopathic individuals.^20^ However, it is difficult to establish a causal relationship because the underlying etiology is unclear, although differences in height at birth suggest that there may be an association with intrauterine growth restriction.

INSL3 levels were higher in cases than in controls, which suggests that the etiology of 46,XY DSDs involves not only normal testosterone secretion but also the functioning of preserved Leydig cells. INSL3 levels may increase from the intrauterine period onwards. Anand-Ivell et al. reported that individuals with ambiguous genitalia (hypospadias and cryptorchidism) had elevated levels of INSL3 in the amniotic fluid in the second trimester, compared with those in controls.^34^ Analyzing the groups separately, the 5ARD2 and PAIS groups showed an increasing trend with regard to INSL3 levels, compared with individuals in the control group. This trend may have been associated with two functional aspects of INSL3: firstly, this hormone may be present at high levels to protect against apoptosis in germ cells,^35,36^ a mechanism that is potentially exacerbated in individuals with 5ARD2 and PAIS, thus resulting in infertility;^36^ and secondly, this increment in INSL3 levels is potentially associated with its induction of steroidogenesis in the context of both relative (5ARD2) and partial (PAIS) androgen insufficiency.^32,37,38,39^

Another potential explanation for this increment in INSL3 levels is Leydig cell hyperplasia. This has been already reported among individuals with 5ARD2 and PAIS at puberty, occurring after LH hyperstimulation through a negative feedback mechanism in 5ARD2, with reductions in DHT and in PAIS, owing to testosterone activity.^33^ However, further research on INSL3 behavior in 46,XY DSD patients is required in order to elucidate this trend.

Multivariate analysis was performed, and no data that would correlate the findings regarding inhibin B, AMH and INSL3 levels with puberty, age and anthropometric data were found, except for weight and BMI. However, there was low explanatory power for these two variables.

One limitation of this study was the small cohort, which may have influenced the statistical power of some of our findings. However, it should be noted that it is not easy to recruit individuals with such rare disorders, through molecular diagnosis at a single center. In addition, the lack of evaluation of testicular histology and its correlation with hormonal levels formed another study limitation, thus further restricting the elucidation of cellular patterns. However, gonadal biopsy is not usually performed for management of 5ARD2 and PAIS.

## CONCLUSIONS

To our knowledge, our study was the first to report that individuals with 5ARD2 have low levels of inhibin B, thus suggesting that this hormone may be a biochemical marker that can differentiate the diagnosis of 5ARD2 from that of other etiologies with a similar clinical presentation (particularly PAIS). Our study was also the first to report that INSL3 levels were higher in patients with 46,XY karyotype, palpable gonads and normal testosterone secretion. However, further evaluation of each etiological group analyzed is necessary. Lastly, for differentiation of these groups, the AMH levels did not show promising results.
